# Further Evaluation of Youth Mental and Behavioral Health in Rural Communities

**DOI:** 10.3390/bs16010014

**Published:** 2025-12-20

**Authors:** Patrick W. Romani

**Affiliations:** Department of Pediatrics, University of Colorado School of Medicine, Aurora, CO 80045, USA; patrick.romani@northwestern.edu

**Keywords:** adolescents, children, mental and behavioral health, rural

## Abstract

Mental and behavioral healthcare (MBH) needs of youth have been increasingly recognized over the past decade. Youth living in rural areas of the United States face a particular challenge in accessing appropriate MBH services. However, the individual needs of communities vary. The current study recruited 32 caregivers, healthcare providers, and educators from four rural communities in one state in the Mountain West region of the United States. These participants responded to questions during a 45–60-min focus group. The goal of the focus groups was to understand (a) how caregivers, healthcare providers, and educators perceive the types of MBH services that are available in their community; (b) what barriers they perceive to accessing satisfactory MBH services; and (c) what strengths they perceive within their community’s MBH system. Results of a thematic analysis of focus group transcripts found common and conflicting themes that likely have an impact on the development of an effective MBH system for rural youth.

## 1. Introduction

Understanding the mental and behavioral health (MBH) needs of youth in rural areas of the United States is a critical public health priority (Graves et al., 2024; Morales et al., 2020). Realities specific to rural living—such as geographic isolation, limited access to healthcare services, socioeconomic disparities, and stigma related to seeking mental healthcare—pose significant challenges to maintaining well-being (Guerrero et al., 2019; Cherry et al., 2017; van den Berk-Clark et al., 2025). Youth in rural areas experience disproportionately high rates of depression, anxiety, substance use, and suicide (Graves et al., 2020). Consequently, there is an urgent need to understand the unique issues surrounding rural youth MBH to drive effective policy and intervention development.

Approximately 20% of the United States’ population lives in a rural community (Morales et al., 2020). Rural communities are typically defined as having fewer than 5000 residents and 2000 housing units (Geverdt, 2019). Living in a small, rural community has notable benefits. For instance, prior research has found that residents often choose rural living because of close community ties, a sense of safety, and a slower pace of life (An et al., 2025). Many rural communities are multigenerational, with residents valuing the support and nurturing environment that comes from being close to family and friends.

Creating a nurturing environment for youth requires modeling supportive relationships, reinforcing prosocial behavior, and limiting opportunities for antisocial behavior (e.g., adult supervision; Biglan et al., 2020). Biglan et al. (2020) demonstrated that nurturing parents and schools foster self-regulation, language and social skill development, academic success, prosocial behavior, and friendships. In contrast, low-quality parenting and schooling—characterized by stress and coercion—can lead to poor self-regulation, aggression, academic failure, peer rejection, depression, and substance use. Despite the many strengths of rural life, conditions such as poverty can disrupt nurturing environments and contribute to MBH concerns (Monatt & Rigg, 2015). Poverty can create coercive environments that hinder self-regulation, increasing the risk of social rejection and association with deviant peer groups. Membership in such groups is associated with early childbearing, drug abuse, depression, antisocial behavior, and ultimately premature death. This cycle illustrates the generational trauma documented in many rural communities.

Although the United States spends at least twice as much per capita on healthcare as most other developed nations, outcomes in health and longevity remain comparatively poor (Biglan, 2018). A stronger mental healthcare system is needed—one that emphasizes caring interactions and compassionate engagement. Social determinants of health continue to erode the development of nurturing communities, and rural areas face particular challenges due to a shortage of mental health professionals, especially those serving youth. Unnecessary barriers and delays in accessing appropriate care further compound the problem (Graves et al., 2024). In addition, concerns about privacy and stigma deter many youth from seeking care. Rural residents often report fears of judgment and confidentiality breaches as reasons for avoiding mental health services (Johansson et al., 2019).

Numerous interventions have been shown to reduce stress in home and school environments and improve MBH outcomes. For example, Ary et al. (1999) demonstrated that family-based therapy promoting youth social, physical, emotional, and behavioral well-being reduced MBH symptoms. Yet, even the most effective interventions can fail when applied at the community level without attention to local context. The phrase “Nothing for us, without us” underscores the necessity of involving rural communities directly in efforts to address MBH. For close-knit rural areas, solutions must be homegrown and grounded in the specific social and environmental realities of the community.

Research documenting the lived experiences of rural community members is critical for understanding MBH in these settings (Garbacz et al., 2022). Qualitative methods, such as thematic analysis of interview data, provide valuable insights into the perspectives of rural residents. This approach has yielded findings that inform policy and intervention development across diverse populations. For example, Garbacz et al. (2022) conducted focus group interviews with caregivers and educators in one rural community, identifying barriers and opportunities for MBH care. Participants emphasized that schools were often the first and most consistent providers of MBH support. Thematic analysis also revealed that limited accessibility to MBH services and family dysfunction created stressful environments for youth. Improved communication between MBH providers and educators was identified as a potential facilitator of more effective intervention delivery. While informative, these findings were limited to a single community. To strengthen rural MBH systems more broadly, research must identify common themes across multiple communities while also recognizing unique contextual differences.

The purpose of the present study was to replicate and extend previous research by using focus groups to examine (a) how caregivers, healthcare providers, and educators perceive the types of MBH services available in their community; (b) what barriers they perceive to accessing satisfactory MBH services; and (c) what strengths they perceive within their community’s MBH system.

## 2. Materials and Methods

### 2.1. Participants and Setting

The present study recruited community members by connecting with local schools, healthcare offices, and family groups from four communities that met the definition of rural as provided by the National Center for Education Statistics (Geverdt, 2019). We targeted these four communities because members of this project’s Community Advisory Committee (CAC) worked or lived in the communities. The four communities had an average population of 4,830 residents. The largest ethnic groups were White (M = 47.9%), White Hispanic (M = 24.4%), and Hispanic (M = 10.3%), with the remaining 17.4% of the population identifying as other ethnicities, such as Black and American Indian and Alaska Native. An average of 28.8% of the community’s residents lived below the poverty line.

The research team first tried to establish communication via e-mail followed by a virtual meeting to explain the purpose of the project. Community members eligible to participate in this study needed to live in one of the four rural communities targeted for this study and be over the age of 13 years at the time of the study. Interested community members were asked to either complete a brief survey in Qualtrics or to e-mail the research team directly to review the consent document.

Over the course of 8 months, we recruited 36 community members from four rural communities in a state in the Mountain West region of the United States. Each community met the definition as being rural based on definitions provided by the National Center for Education Statistics (Geverdt, 2019). Please see Table 1 for a description of study participants. Of the 36 participants, 8 identified as caregivers/parents, 12 identified as healthcare workers (e.g., pediatrician, licensed professional counselor), and 16 identified as educators (e.g., school social worker, school psychologist). 25 of the 36 participants identified as White with the remaining 11 participants identifying themselves as Hispanic. 32 of the participants identified as female with the remaining 4 participants identifying themselves as male.

Participants engaged in focus group discussions among groups of 2–5 other individuals. Most focus group meetings occurred in the conference room of the elementary school in each of the four communities. A smaller portion of the focus group meetings occurred via a virtual meeting platform (Zoom).

### 2.2. Measures

Prior to beginning the current study, the researcher formed a Community Advisory Committee (CAC). The CAC comprised four community members that worked in education, mental healthcare, or had lived experience with mental illness or caring for a child with mental illness. Using our collective experiences, we developed a 14-question interview guide. An example question asked to participants was, “What are the biggest barriers to children receiving mental and behavioral healthcare services in your community?” The interview guide contained items regarding the major research questions for this work.

### 2.3. Procedure

This study recruited caregivers, educators, and students to participate in focus groups. We conducted eight focus groups with an average of 2.4 participants (range, 2–5 participants). The lead author conducted the focus groups with assistance from the rest of the CAC. We recorded focus group content for subsequent transcript and review. Focus groups lasted approximately 30–45 min.

The lead author started each focus group by reviewing the informed consent document. This process also included a description of the purpose of the focus group. After participants signed the informed consent document, the lead author started a voice recording to capture the focus group discussion. During the focus group, the lead author asked questions regarding (a) what MBH services are available in the community, (b) what expectations are for therapy from the parent, educator, and youth point of view, (c) accessibility of MBH services, (d) strengths of the MBH system in the community, and (e) opportunities for the MBH system in the community. The lead author asked clarifying questions as needed but let the focus group participants lead the conversation. At the end of the focus group, the voice recording was terminated, and participants were thanked for their time. Participants received $50 cash as compensation for participation.

This study was situated within a qualitative interpretivist/constructivist paradigm, which emphasizes understanding how participants construct meaning through their lived experiences. A utilization-focused approach to transcript analysis was therefore employed to prioritize findings that are directly applicable to practice and meaningful to key stakeholders.

### 2.4. Analysis

**Positionality.** Our CAC consisted of four members. The primary investigator identified as a faculty member at a state university who had interests in MBH in rural communities. The second member of the CAC led a team of MBH clinicians within a local health system in the targeted rural communities. She moved to the rural community with family. Another member of the team that assisted with interpreting parent, educator, and student data represented the rural community’s local health department. She grew up in the rural community. The last member of the research team that supported the analysis of parent, educator, and student data moved to the rural community with family. She worked as a community liaison for community engagement practices at a state university. All members of the research team had significant interest in youth MBH in their rural community.

**Unitization.** There were two coders for the current project, and they analyzed all participant responses during their focus group interviews. Participant responses during each focus group interview were transcribed using iTranscribe. The coders reviewed all transcripts to correct errors that may have occurred. The coders developed a unitization manual after studying all transcripts. The manual contained definitions for key ideas among each of the three research questions and examples and non-examples of each definition and when it would be categorized into each of the research questions. Intercoder agreement was assessed by having a third research team member code all responses using the same manual. The overall kappa agreement was 0.86 (range, 0.80–0.88) for the caregiver interviews, 0.82 (range, 0.76–0.88) for the educator interviews, and was 0.88 (range, 0.84–0.94) for the healthcare provider interviews. Disagreements were discussed amongst the coders until they were able to come to a consensus on 100% of units.

**Categorization.** After the unitization process, the same two coders reviewed the units and assigned the three research questions. The coders evaluated the units for themes to develop minor categories. Minor categories were those that three or more participants endorsed in similar ways (Garbacz et al., 2022). An independent coder reviewed 33% of the units sorted into each minor category. That is, 33% of units for each minor category were reviewed. The overall kappa agreement was 0.88 (range, 0.82–0.90) for the caregivers, 0.84 (range, 0.8–0.89) for the educators, and 0.82 (range, 0.76–0.87) for the healthcare providers. Disagreements were discussed amongst the coders until they were able to come to a consensus on 100% of units.

## 3. Results

Analysis of focus group responses revealed both major and minor categories for caregivers, educators, and healthcare providers. We organize the findings below by research question. We will describe the major and minor categories by describing the number of units from each category along with example quotations. Please see Table 2, Table 3, Table 4, Table 5, Table 6, Table 7, Table 8, Table 9 and Table 10 for more information.

**RQ1.**  *What do caregivers, educators, and students report as the available MBH services in their community?*

**Caregiver Perspectives.** Caregivers identified a total of 29 units. The major categories were: (a) geographic and provider limitations and (b) mental health stigma. Please see Table 2 for a complete description of caregiver major and minor categories.

***Geographic and Provider Limitations.*** We identified geographic and provider limitations as the largest major category (n = 18). The following minor categories were identified, too: (a) overwhelmed healthcare providers (n = 8) and (b) long-distance travel (n = 10). The caregivers made comments similar to, “The people that we have to do the work are completely overworked and overwhelmed,” that supported the minor category of overwhelmed healthcare providers. The remaining minor category described barriers to MBH services. For example, caregivers made comments similar to, “…people can’t afford to drive and take off work” indicating that financial and job-related stressors adversely impact caregiver ability to bring their children to MBH appointments. Additionally, and this may relate to overwhelmed healthcare providers too, caregivers described the importance of having more MBH services in their schools.

***Mental Health Stigma.*** The remaining 11 units were sorted into the major category of mental health stigma. Six units fell into the “dual relationships with healthcare providers” and the other five units were sorted into the “lack of community openness to MBH discussion.” Related to the former minor category, caregivers described numerous concerns related to seeing therapists at community events, like visiting the grocery store or at a school-based sports event. For example, one caregiver reported, “I could see the therapist, nurse, whoever at the grocery store or at church.” With respect the latter minor category, caregivers acknowledged MBH symptoms related to depression, anxiety, and trauma. However, they also reported that stigma around mental health drives community openness to seeking services. For example, one caregiver reported that they, “…keep discussions in the family.” In other words, they do not talk about their MBH symptoms with others in the community.

**Educator Perspectives.** Educator responses led to the identification of 27 total units and two major categories (Table 3). The major categories were “need for more family engagement” (n = 15) and “difficulties forming partnerships between school and community providers” (n = 12).

***Need More Family Engagement.*** Related to the first major category (i.e., family engagement), “failure to attend sessions or follow-up with paperwork” (n = 8) and “families are essential for improvement” (n = 7) emerged as the minor categories. The teachers recognized disruptive behavior and internalizing symptoms, like depression, as contributing to youth MBH in their community. There seems to be therapists in their community that address these concerns. However, they also acknowledged how caregiver involvement likely benefits the entire process of starting and conducting MBH therapy. For example, they noted concerns like, “It’s taking a lot to work on the family needing support to engage with the agency” and “The struggle is more frequently on the end of the parent…”

***Difficulties Forming Partnerships Between School and Community Providers.*** The minor categories for this major category are “regulatory obstacles” (n = 6) and “advocate support” (n = 6). Several educators felt that the process of obtaining consent to interact and share information with outpatient providers led to unnecessary delays to treatment. Several of the educators also referenced the value of educational advocate support and how their availability can be difficult to predict.

**Healthcare Providers.** We identified 23 units from healthcare providers which formed two major categories: (a) referral process for services (n = 14) and (b) geographic limitations to service availability (n = 9).

***Referral Process for Services.*** The first major category led to the development of two minor categories: (a) organization-specific requirements (n = 7) and (b) unclear school-community partnership (n = 7). The healthcare providers noted challenges with establishing services community-based MBH therapy. For example, one healthcare provider noted that they, “Don’t make referrals because it requires some type of form…” As it relates to the second minor category, the healthcare providers questioned the effectiveness of the local provider’s collaborations. In one example, a healthcare provider expressed concerns for how the shortened school week created a backlog of patients wanting to be seen on Friday, the day off.

***Geographic Limitations to Service Delivery.*** The second major category described concerns regarding geographic barriers to receiving MBH services. Two minor categories emerged from these data: (a) dependence on overwhelmed local providers” (n = 5) and (b) barriers to services (n = 4). The healthcare providers noted a lack of responsiveness from community providers that affected their confidence in an already small pool of MBH providers in the area. The providers also noted that feelings of “isolation” and “difficulties affording travel” contributed to the geographic limitations in the area.

**RQ2.**  *What do caregivers, educators, and healthcare providers indicate are the biggest barriers to receiving satisfactory MBH services in the region?*

**Caregiver Perspectives.** A total of 31 units were identified for the caregiver responses to this research question. We categorized these units into two major categories: (a) stigma and difficulties seeking help (n = 19) and (b) job support (n = 12). Please see Table 5 for these data.

***Stigma and Difficulties Seeking Help.*** We divided this major category into two minor categories: (a) not sure what is developmentally appropriate or not (n = 9) and (b) need advocacy to normalize MBH symptoms (n = 10). Related to the first minor category, caregivers noted difficulties understanding typical child development. For example, one caregiver reported, “Nothing really prepares you to under what’s considered to be normal…” The second minor category describes parent’s interest in organizing to support others acknowledge MBH symptoms. For example, “More people need to be OK not being OK.”

***Job Support.*** This major category was divided into the “variability in employer expectations (n = 7) and “corporate versus small business support” (n = 5) minor categories. Parents reported variability in how their employers responded when they needed to take time off for their child’s MBH. If they worked for a large corporation, like Wal Mart, they reported more difficulties attaining time off for MBH appointments. Caregivers also reported that they also felt badly taking time off work for their child’s MBH. When they took time off work, another colleague needed to cover, or the team would work with one less employee.

**Educator Responses.** We identified 28 units for the educators. This resulted in two major units: (a) stigma and barriers to MBH support (n = 18) and (b) local context and community challenges (n = 10). Please see Table 6 for these data.

***Stigma and Barriers to MBH Support.*** We identified two minor categories for this major category: (a) improved stigma around MBH (n = 11) and (b) stigma as a problem (n = 7). Interestingly, the educators reported mixed feelings regarding stigma and barriers to MBH support. Some educators reported comments like, “I feel like stigma is getting better…” In contrast, others would discuss the lack of acceptance for MBH symptoms.

***Local Context and Community Challenges.*** We divided this major category into two minor categories: building relationships (n = 5) and changes in the ethnic make-up of the community (n = 5). Several educators felt that they could make progress with community members by building relationships with them. For example, one educator noted that focusing on fathers may be an effective strategy to reduce barriers. Others noted that changes to the ethnic make-up of the community have contributed to barriers. For example, an increasing Latin presence has been challenging because, as they reported, the Latin culture keeps symptoms related to MBH to themselves.

**Healthcare Providers.** A total of 14 units were identified from the healthcare provider’s discussions. This led to the development of two major categories: (a) stigma around mental health (n = 7) and (b) need for appropriate services (n = 7). Please see Table 7 for these data.

***Stigma Around Mental Health.*** The healthcare provider’s comments were divided into two minor categories: stigma as a barrier (n = 4) and time to build rapport and trust (n = 3). The healthcare providers reported that stigma had regressed over time and that stigma was “a part of the culture.” The healthcare providers also reported that working to build trust and rapport could support the barriers in place. For example, one provider said, “I think for parents, it’s trust…”

**RQ3.**  *What do caregivers, educators, and healthcare providers perceive as strengths of the MBH system in their area?*

**Caregiver Responses.** We identified 34 units based on caregiver responses, which led to two major categories: school-based services (n = 19) and expansion and diversification (n = 15). Two minor categories came out of the expansion and diversification major category (i.e., respite services [n = 8]; specialized therapies [n = 7]).

***School-Based Services.*** Minor categories produced from the school-based services major category were (a) appropriate curriculum (n = 9) and (b) increased educator support (n = 10). Caregivers made comments about the importance of schools for delivering MBH services as well as making appropriate therapeutic resources available. With respect to respite services and educator support, caregivers note the difficulties with maintaining resiliency. Since caregivers and educators spend considerable time with the area’s youth, the caregivers reported the importance of finding resources for respite.

***Expansion and Diversification.*** Under the expansion and diversification major category, increased access to respite workers was noted to be important as caregivers need healthy boundaries to support their own mental health. Caregivers also indicated that the MBH providers currently in their community need additional, perhaps more specialized, training in order to work best with most kids.

**Educator Responses.** A total of 29 units were identified based on the educator’s responses. We divided these units into two major categories: (a) enhanced school-based support (n = 16) and (b) coalescence around areas of need (n = 13).

***Enhanced School-Based Support.*** Two minor categories emerged from this major category: new resources for students (n = 8) and grant-funded programs (n = 8). Educators made note that of the newer resources being introduced to school settings. For example, one educator reported she felt impressed by the mindfulness classes integrated into the school system. Educators also indicated that grant-funded programs help enhance the types of services offered in the school.

***Coalescence Around Areas of Need.*** The minor categories were determined to be (a) extracurricular options (n = 6) and (b) serving underrepresented student groups (n = 7). Many educators discussed the importance of extracurricular activities to support better youth MBH. The educators also commonly reported wanting more specialized services for LGBTQIA+ students. One educator recognized how difficult it can be for youth with different sexual orientations to focus on day-to-day responsibilities.

**Healthcare Provider Responses.** A total of 36 units were produced from the healthcare provider’s responses. 19 units coalesced around the major category of integrating services into schools, and 17 units coalesced around the second major category of evaluation of parental awareness and involvement.

***Integrating Services into Schools.*** Two minor categories emerged: improved care coordination (n = 10) and improving school-based services (n = 9). A number of healthcare providers reported that schools are improving in their efforts to coordinate care. Additionally, the healthcare providers reported that the curriculum and teaching methods schools are using to work on youth MBH are positive.

***Evolution of Parental Awareness and Involvement.*** The healthcare providers noted that positive changes to MBH stigma are occurring in the community. This minor category was called “positive changes to MBH stigma” (n = 8). The second minor category was increased awareness and appreciation for MBH symptoms (n = 9). Providers noted that caregivers appear to be more aware of when their kids need help. Caregivers also appear to be reaching out for help, per their report.

## 4. Discussion

The current study had three primary aims: to examine caregivers’, educators’, and healthcare providers’ perceptions of (a) the types of MBH services available in their community, (b) barriers within their MBH system, and (c) strengths of the MBH system in their community. Thirty-two community members shared their perspectives to help answer these questions. This study extends the existing literature, which has primarily focused on caregiver and educator perspectives (Garbacz et al., 2022), by also including healthcare providers such as licensed professional counselors, social workers, pediatricians, and nurse practitioners.

Across the three stakeholder groups, participants consistently identified trauma, depression, suicidality, and substance abuse as the primary MBH concerns in their community. Caregivers and educators, in particular, emphasized the importance of addressing disruptive behaviors, including tantrums, aggression, and noncompliance. These findings are consistent with prior studies that have documented similar challenges across different regions of the country (Bitsko et al., 2022; Morales et al., 2020). Several factors may explain why these MBH concerns are prevalent in these rural communities. Educators highlighted the lack of recreational activities for youth, a reality likely tied to the community’s small population as well as environmental factors, such as narrow roads and sidewalks (Kegler et al., 2022; Yousefian et al., 2009). Without structured recreational outlets, youth may be more likely to engage in alternative, riskier activities such as substance use. For example, Rigg and Johnson (2022) used data from 65,248 adolescents involved with the Florida Department of Juvenile Justice. They found that 2.3% of the sample met criteria for opioid misuse and that engagement in extracurricular activities reduced the risk for opioid misuse by 36%. In addition, the absence of recreational opportunities often keeps youth at home, where strained family dynamics may increase conflict and trauma exposure.

Given these realities, all respondents emphasized the importance of youth MBH services. However, geographic barriers significantly limited access to care. Families often struggle to attend appointments because of travel costs (e.g., fuel expenses) or because services are simply unavailable nearby. Caregivers also identified stigma as a major deterrent to seeking care. When community members are already uncertain about engaging in MBH services, the additional burden of travel and logistics may further reduce service use. These sentiments are similar to those reported in other published research (e.g., Crumb et al., 2019).

Interestingly, perspectives on stigma varied across groups. Educators suggested that stigma around MBH may be improving, while caregivers continued to view it as a major challenge. Healthcare providers were divided in their views. This lack of consensus may pose challenges: if educators and providers assume stigma has lessened, their recommendations may not adequately reflect caregivers lived experiences.

Two facilitators of an effective MBH system emerged in these communities. First, schools were widely recognized as central hubs for youth MBH services. In rural areas, schools often represent the first and sometimes only point of contact for MBH support (Garbacz et al., 2022). School-based services may include counseling, social-emotional learning programs, and early intervention supports delivered directly to students. For many rural youth, schools provide their most accessible and trusted source of care. Integrating MBH services into schools can help reduce barriers to access. By offering care in a familiar and less stigmatizing setting, schools may increase engagement among students who might otherwise avoid treatment (Weist et al., 2019). Teachers, counselors, and administrators play key roles in identifying early signs of concern and facilitating referrals to appropriate providers. However, rural schools often face obstacles such as limited funding, understaffing, and insufficient training for school personnel (Michael et al., 2023). Many schools lack dedicated MBH professionals and rely on existing staff without specialized training. Addressing these gaps requires investment in school-based programs, professional development for educators, and stronger partnerships between schools and community health providers to ensure continuity of care. Strengthening the capacity of schools to deliver MBH services could significantly improve youth outcomes in rural communities. A second potential facilitator was caregiver engagement, though findings here were mixed. Some respondents noted that caregivers were increasingly involved in establishing and maintaining MBH services, while others reported less consistent engagement. Educators emphasized that relationship-building with parents was essential, noting that some caregivers became more receptive to services when professionals demonstrated patience and support during the decision-making process.

This study is not without limitations. First, it was conducted in a single rural community in the Mountain West region of the United States, which limits generalizability. Similar issues that may affect generalizability include the relatively high proportion of focus group participants with a college degree or greater as well as the relative brevity of the focus group sessions (i.e., 30–45 min). Nonetheless, findings align with a growing body of research documenting similar MBH concerns among rural youth, suggesting consistency across contexts (e.g., Garbacz et al., 2022). Second, the CAC could have been more inclusive of individuals working with youth experiencing MBH concerns. For example, we could have included nurses or paraprofessionals from a school setting. This should be considered during similar work in the future. Third, qualitative coding methods may have inadvertently overlooked important information, as is always a risk when interpreting interview data. Future research should address these limitations by examining broader samples of rural communities and refining qualitative methods to capture a more comprehensive picture of community needs.

## Figures and Tables

**Table 1:** Demographic information.

	Caregiver (n = 8)	Educator (n = 16)	Healthcare Provider (n = 12)	Total (n = 36)
Race/ethnicity				
White	6 (75%)	11 (68.8%)	8 (66.7%)	25 (69.4%)
Hispanic/latino	2 (25%)	5 (31.3%)	4 (33.3%)	11 (30.6%)
Gender				
Female	7 (87.5%)	15 (93.8%)	10 (83.3%)	32 (88.9%)
Education				
Less than HS diploma	0	0	0	0
HS diploma	3 (37.5%)	0	0	3 (8.3%)
GED	0	0	0	0
Some college	0	0	0	0
College degree	3 (37.5%)	10 (62.5%)	0	13 (36.1%)
Some graduate coursework	0	3 (18.8%)	1 (8.3%)	4 (11.1%)
Master’s degree	2 (25%)	3 (18.8%)	9 (75%)	14 (38.9%)
Doctoral degree	0	0	2 (20%)	2 (5.6%)

**Table 2:** Caregiver responses to RQ1.

Major Categories	Minor Categories	Participant Quotes
Geographic and provider limitations (18)	Overwhelmed healthcare providers (8)	“There are only a few therapists and they are overwhelmed.”
		“A lot of people cannot afford to drive and take off work.”
	Barriers to MBH services (10)	“We need more at school. School is convenient. The kids are there anyways.”
Mental health stigma (11)	Dual relationships with healthcare providers (6)	“I could see the therapist, nurse, whoever at the grocery store or at church.”
	Lack of community openness to MBH discussion (5)	“…keep discussions in the family.”

**Table 3:** Educator responses to RQ1.

Major Categories	Minor Categories	Participant Quotes
Need for more family engagement (15)	Failure to attend sessions or follow-up with paperwork (8)	“There are two agencies that have long waitlists.”
		“It’s taking a lot of work on the family needing support to engage with the agency”
	Families are essential for improvement (7)	“The struggle is more frequently on the end of the parent…”
Difficulties forming partnerships between school and community providers (12)	Regulatory obstacles (6)	“Because of confidentiality, it kind of makes it difficult for us to know who gets what, when, and how much”
	Advocate support (6)	“Family helper drove them to the session and the therapist let the parent work in the office while she met with their kid”

**Table 4:** Healthcare provider responses to RQ1.

Major Categories	Minor Categories	Participant Quotes
Referral process for services (14)	Organization-specific requirements (7)	“We don’t make referrals because it requires some type of form. We give patients the phone number and advise them to call and self refer.”
	Unclear school-community partnership (7)	“Kids are booking on Fridays because of the four day school week. I wish they could be seen other times.”
Geographic limitations to service availability (9)	Dependence on overwhelmed local providers (5)	“I didn’t get an answer and haven’t used them as much.”
	Barriers to service (4)	“A little bit of isolation, but, you know, we do what we can.”

**Table 5:** Caregiver responses to RQ2.

Major Categories	Minor Categories	Participant Quotes
Stigma and difficulties seeking help (19)	Not sure what is developmentally appropriate or not (9)	“Nothing really prepares you to understand what’s considered to be normal. So, when are you dropping the ball?”
	Need advocacy to normalize MBH symptoms (10)	“More people need to be OK not being OK.”
Job support (12)	Variability in employer expectations (7)	“If I had to leave early, I’m sure they would [let me]. It’s just a lot more difficult because their short now.”
	Corporate versus small business support (5)	“Big corporations don’t care if your child has a disability. Their thought is the dollar sign. My store is family oriented and still want me there.”

**Table 6:** Educator responses to RQ2.

Major Categories	Minor Categories	Participant Quotes
Stigma and barriers to MBH support (18)	Improved stigma around MBH (11)	“I feel like stigma is getting better around mental health.”
	Stigma as a problem (7)	“Our community struggles with being as accepting or as progressive as a lot of other communities.”
Local context and community challenges (10)	Building relationships (5)	“The dad’s are starting to say like, I wish I wouldn’t known. I could’ve done…It just takes building relationships.”
	Changes in ethnic make-up of community (5)	“We also have a very, very strong Latin presence in the community now. I think that culture has a lot to do with not getting mental health services.”

**Table 7:** Healthcare provider responses to RQ2.

Major Categories	Minor Categories	Participant Quotes
Stigma around mental health (7)	Stigma as a barrier (4)	“The stigma of mental health. I think we’re in a slightly worse place than we were a year ago.”
		“I think there’s a real stigma around mental health…it’s just not part of the culture.”
	Time to build rapport and trust (3)	“I think for parents, it’s trust. They don’t really trust someone to work with their child necessarily.”
		“I think it would be tremendous if we had more folks go to the house to get that relationship started.”
Need appropriate services (7)	Short versus long-term therapy (4)	“I wish we were still providing long-term therapy for our patients.”
	School-based support (3)	“I wish they knew that really good social emotional curriculum can get them [the student] through the rest of their day.”

**Table 8:** Caregiver responses to RQ3.

Major Categories	Minor Categories	Participant Quotes
School-based services (19)	Appropriate curriculum (9)	“Schools make it [therapy] more available to kids. They need the right stuff”
		“In our area, schools are a big hub for mental healthcare services.”
	Increased educator support (10)	“I think that one way to support mental health for kids here is supporting the people that support them, which is the teachers in the schools.”
Expansion and diversification (15)	Respite Services (8)	“Parents need a break. We need respite workers badly.”
	Specialized therapies (7)	“I would like to see people trained to work with my specific kid.”

**Table 9:** Educator responses to RQ3.

Major Categories	Minor Categories	Participant Quotes
Enhanced school-based support (16)	New resources for students (8)	“The classes we’re offering, like the mindfulness that wasn’t in schools when I was in school. It’s really neat. I’m pretty impressed by it.”
	Grant-funded programs (8)	“The grant is for things done through the schools…marijuana education, science of suicide, social-emotional learning.”
Coalescence around areas of need (13)	Extracurricular options (6)	“We need a rec center or something, something that’s not just sports.”
	Serving underrepresented student groups (7)	“We need more mental health support for LGBTQIA+ students. Sometimes I don’t even know how they focus on school.”

**Table 10:** Healthcare provider responses to RQ3.

Major Categories	Minor Categories	Participant Quotes
Integrating services into schools (19)	Improved care coordination (10)	“I think school is pretty good about getting a high overview. I am getting to know more of the school therapists.”
	Improving school-based services (9)	“I hear schools are getting new ways of teaching kids about stress. That is good.”
Evolution of parental awareness and involvement (17)	Positive changes to MBH stigma (8)	“I grew up on a farm, graduated high school from here and left and came back. I would say stigma is getting better. More people say they want to go see a counselor.”
	Increased awareness and appreciation for MBH symptoms (9)	“I think parents are reaching out more. Probably not to the extent that really needs to happen but I think it’s improvement.”
		“Parents are more aware of when their kids need help.”

## Data Availability

The data that support the findings of this study are not publicly available due to privacy concerns. The data are, however, available from the author upon reasonable request.
